# Contrast Enhanced Abdominal Ultrasound in the Assessment of Ileal Inflammation in Crohn’s Disease: A Comparison with MR Enterography

**DOI:** 10.1371/journal.pone.0136105

**Published:** 2015-08-31

**Authors:** C. S. Horjus Talabur Horje, R. Bruijnen, L. Roovers, M. J. M. Groenen, F. B. M. Joosten, P. J. Wahab

**Affiliations:** 1 Department of Gastroenterology, Rijnstate Hospital, Arnhem, the Netherlands; 2 Department of Radiology, Rijnstate Hospital, Arnhem, the Netherlands; 3 Department of Epidemiology and Statistics, Rijnstate Hospital, Arnhem, the Netherlands; CWRU/UH Digestive Health Institute, UNITED STATES

## Abstract

**Background and Aims:**

To prospectively examine the feasibility and accuracy of Contrast Enhanced Ultrasound (CEUS) in the assessment of Crohn’s disease (CD) activity in the terminal ileum in comparison to Magnetic Resonance Enterography (MRE), using endoscopy as a reference standard.

**Methods:**

105 consecutive patients with alleged clinically active CD were assessed by MRE and CEUS. CEUS of the terminal ileum was performed using an intravenous microbubble contrast enhancer. Accuracy values of CEUS and MRE for the presence of active terminal ileitis were evaluated using the Receiver Operating Characteristic method, using endoscopic findings as a reference standard. Sensitivity and specificity values of MRE and CEUS were compared by the McNemar test.

**Results:**

CEUS was feasible in 98% of patients, MRE in all. Optimal diagnostic accuracy in CEUS was obtained at a peak intensity value of 10%, showing 100% sensitivity, 92% specificity and an accuracy of 99% in demonstrating ileal mucosal inflammation. For MRE, overall sensitivity, specificity and accuracy were, 87%, 100%, and 88%, respectively. CEUS and MRE were highly correlated in assessing length and wall thickness of the terminal ileum. CEUS identified 11 of 16 MRE-detected strictures, but no fistulae.

**Conclusion:**

The accuracy of CEUS is comparable to that of MRE in the assessment of active, uncomplicated terminal ileal CD and therefore a valuable bedside alternative to MRE in the follow-up of these patients.

## Introduction

Crohn’s disease (CD) is a chronic transmural inflammatory disease of the gastrointestinal tract characterized by a widespread variation in severity and relapsing episodes of disease activity. Exclusive ileum disease is observed in approximately 30% of patients, whereas the ileocecal segment is involved in more than 50% of all CD patients [1]. Assessment of the severity of CD-associated inflammatory activity is used to monitor therapeutic results and plan further medical or surgical therapy. Clinical markers and laboratory tests are suboptimal indicators of disease activity [2, 3]. Additional methods to reliably estimate disease activity include endoscopy and radiological imaging techniques. Ileocolonoscopy is considered the gold standard for CD disease activity in the colon and terminal ileum, allowing tissue sampling. This however is an invasive and, from patients’ perspective, burdensome method [4]. Complete visualization of the small bowel and of extramural disease manifestations may be performed by CT Enterography and MR Enterography (CTE and MRE) [5–7]. These two techniques have a similar diagnostic accuracy for CD disease extension and activity, as reported by Panes et al [8]. MRE is not widely available as it is a rather costly technique, whilst repeated CTE induces cumulative ionizing radiation exposure, particularly important in this group of relatively young patients [9–11]. The sensitivity of grey scale ultrasound (US) for the detection of CD disease activity of the terminal ileum ranges from 63% to 100%, with a specificity in the range of 77–100% [8, 12–15]. Contrast enhanced ultrasound (CEUS) with new intravenous contrast agents allows visualisation of bowel wall perfusion. Quantitative evaluation of enhancement during CEUS improves the accuracy of ultrasound for the presence and severity of active inflammation in ileal CD [16–22]. Ultrasound is less suitable in the assessment of CD activity located in the large bowel, because of the limited ability to accurately localise the affected bowel segments.

The diagnostic value of bowel wall enhancement with CEUS in the evaluation of CD disease activity, in comparison to MRE, was assessed in two studies [23–24]. Both of these used clinical disease activity scores as a reference, showing no correlation with either contrast enhancement by CEUS or by MRE. Therefore, these studies did not provide a clear assessment of the diagnostic value of CEUS as compared to MRE in daily clinical practice.

The aim of this study was to assess the feasibility and accuracy of CEUS in the detection of CD activity in the terminal ileum, as compared to MRE, using endoscopy as a reference standard for active inflammation.

## Materials and Methods

### Study population

From October 2009 to December 2012 a total of 107 consecutive adult patients with established active CD, who attended the Department of Gastroenterology of a large regional hospital (Rijnstate Hospital, Arnhem, NL), were prospectively enrolled in the study. The initial diagnosis of CD located in the terminal ileum, colon or both was confirmed in all patients according to the usual clinical, endoscopic and histological criteria [25]. The inclusion criteria were: age over 18 years and a clinical indication for an ileocolonoscopy, to assess localization, extent and severity of the presumptive activity of CD (primary diagnosis, clinical relapse or recurrence). Patients who did not agree to participate in the study were excluded from the study, as were pregnant women. Further exclusion criteria were: contraindications for MRE such as claustrophobia, MR-unsafe devices and implants, renal insufficiency and conditions potentially related to the toxicity of Sonovue (Bracco, Italy); also a history of ischemic coronary disease and cardiac failure.

The clinical disease activity at inclusion was scored using the Harvey Bradshaw Index [26] and blood samples were taken for the measurement of C-reactive protein (CRP).

The study was performed according to the principles of the Declaration of Helsinki. Approval of the regional ethics committee for medical research from Arnhem-Nijmegen, in The Netherlands (CMO Arnhem-Nijmegen) was obtained on September 2009 and all patients provided written informed consent.

### Endoscopy

All patients underwent endoscopy under conscious sedation. The ileocolonoscopy was performed after standard bowel preparation with 4 liter of polyethylene glycol electrolyte solution. The disease activity was scored using the Simple Endoscopy Score for CD (SES-CD) [27], adjusted to score ileal disease activity. The SES-CD score was originally designed by Daperno et al. to score the mucosal disease activity in Crohn’s disease, through the whole colon and the terminal ileum for the length that has been examined by endoscopy. The four variables selected were: the size of ulcers, proportion of ulcerated surface, the surface with any other lesions and the presence of stenosis. Each variable was scored from 0 to 3 in each of the 5 segments (ileum, right colon, transverse colon, left colon, rectum) with a maximal total score for the SES-CD of 48. When these variables are only scored for the terminal ileum the maximal score is 12. A score of 0 equals no endoscopic lesions and a score of 1 implies the presence of erythematous mucosa over less than 50% of the surface without any aphthous lesions or ulcerations. Before the start of the study, we defined a score of 0–1 in the terminal ileum, as no active inflammation and a score of 2 or higher as active inflammation.

### Magnetic Resonance Enterography

Within a period of maximally two weeks from the reference ileocolonoscopy and before any therapeutic changes, the patients underwent MRE followed by CEUS on the same day.

The MRE studies were performed according to a standardized protocol. Patients fasted for at least 6 hours and were asked to drink 1.0 liter of a water-based oral preparation fluid containing 2,5% mannitol and 0,5% locust bean gum, in a time span of 45 minutes before the MRI, without any particular preparation of the colon. MRI scans were performed with the patient in prone position, using a body coil on a 1.5T MRI unit (Intera 1.5 T, Philips medical systems, Best, Holland). All patients were given butylscopolamine 20 mg intravenously, as intestinal motility inhibitor immediately before the examination to avoid bowel peristalsis artifacts. Patients were scanned in the prone position to avoid wall movement of the abdomen.

The scanning protocol included a coronal breath-hold T2-weighted single-shot (SSh) turbo spin-echo and a coronal T2 breath-hold balanced fast field echo (FFE). The field of view of these T2 weighted studies was the whole abdomen, from the xyphoid process to the pubic bone. Slice thickness was 5 mm consecutive, which resulted in an average of 30 images in the coronal plane. After 60 seconds of manual administration of the intravenous contrast medium (Gadoteric acid, Dotarem) (0.5 mmol/ml) 0.2 ml per kg body weight), T1 weighted sequences were applied in the transverse and coronal plane with a fat saturation spectrally attenuated inversion recovery (SPAIR). The terminal ileum was the center of the field of view of these last three sequences.

The MRE images were analysed by two experienced abdominal radiologists. They were aware of the diagnosis of CD and blinded for clinical, endoscopic and CEUS data. Parameters scored included the length of affected bowel, wall thickness measured at the point of maximum diameter of the affected small bowel wall, stratified wall appearance and perivisceral fibrofatty proliferaton, stenosis, fistulae, abscesses and enhancement after administration of gadolinium (Table 1). Stenosis was defined as a narrowing of a thickened bowel segment with a proximal area of dilation (> 3.0 cm). Fistulae were defined as tubular tracts with high signal intensity, between two small bowel loops. Abscesses were described as a fluid collection, with or without associated air and a well defined wall.

**Table 1:** Radiological parameters assessed by MR Eenterography (MRE), Grey scale Ultrasound (US) and Contrast enhanced Ultrasound (CEUS).

Radiologic parameters	MRE	Grey scale US
Wall thickness	+	+
Length	+	+
Stratified wall appearance	+	+
Perivisceral fibrofatty proliferation	+	+
Stenosis	+	+
Fistulae	+	+
Abscesses	+	+
	MRE	CEUS
Maximum Enhancement	+	+
Time to peak	-	+
Regional Blood Volume	-	+

Mural contrast enhancement was considered present if there was a clear visual enhancement of a small bowel segment compared to adjacent loops of the terminal ileum, at visual assessment.

### Grey scale Ultrasound and Contrast enhanced Ultrasound

Ultrasound was performed with a Philips US device (Philips IU22), using a convex 3–5 MHz probe. All studies started with a grey scale US examination to find the terminal ileum and assess wall thickness, induration of surrounding fatty tissue and enlarged lymphnodes. Stenosis and length of the pathologic bowel wall was estimated, as well as complications such as abscess formation and fistulae. The thickest segment of the terminal ileum was identified and used to perform analysis of contrast enhancement. We injected the second generation, ultrasonic contrast agent (SonoVue, Bracco, Milan, Italy) as a bolus of 2.5 ml through a three-way 20-gauge catheter in an antecubital vein, followed by a bolus of 10 ml saline solution (0.9% NaCl) [28]. CEUS was performed with a 7.5MHz linear probe and contrast-tuned technology, based on a low mechanical index and a real-time scan to ensure the preservation of the contrast agent. A low acoustic power setting was used, expressing a low mechanical index (MI), 0.09–0.14, of a 7.5 MHz linear probe.

Disease parameters analyzed during the grey scale US included identification of the terminal ileum, wall thickness measurement at the maximal diameter of the affected terminal ileum (pathological mural thickening defined as a wall thickness above 3 mm), evaluation of the affected bowel length, stratified wall appearance, perivisceral findings such as creeping fat and stenosis, fistula or abscess (Table 1). Stenosis was described as narrowing of a thickened, rigid bowel lumen with a proximal area of dilation (> 3.0 cm). Fistulae were defined as tubular hypoechoic tracts between two small bowel loops. Abscesses were described as a fluid collection, with or without associated air and a well defined wall [19].

**Fig 1:**
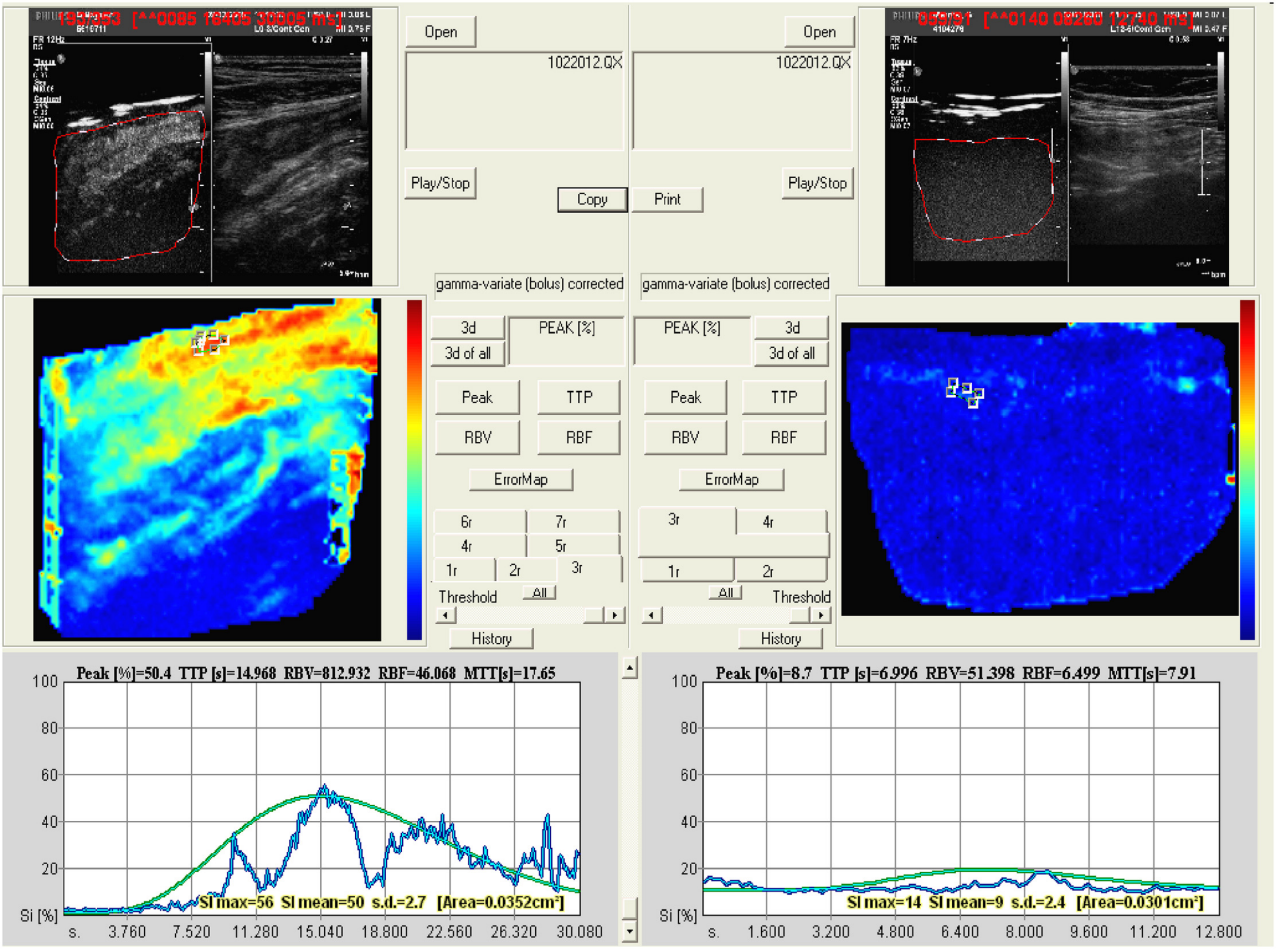
Perfusion map obtained by Qontrast software. Left side: contrast uptake based on increased microvascularization of the terminal ileum in a patient with severe endoscopic disease activity. Right side: contrast uptake of the terminal ileum in a patient with normal endoscopic appearance.

CEUS images were assessed directly after injection of the contrast agent, by recording a videoclip at the level of the most affected terminal ileum loop. After primary imaging, quantification software Qontrast (Bracco, Italy) was applied to obtain contrast-enhanced sonographic perfusion maps, allowing immediate evaluation of the peak intensity (PI), time to peak (TTP), and the regional blood volume (RBV), being proportional to the area under the curve (Fig 1). These parameters, measured within the region of interest (ROI) selected by the operator, have formerly been described as indicators for disease activity [16–19]. Qontrast software is able to quantify the contrast enhancement from a sequence of the perfusion frames and to generate a chromatic map that allows immediate evaluation of the perfusion properties of the selected region (region of interest, ROI). The quantification procedure is being performed for each pixel encompassed by the ROI on the frame sequence. The ultrasound video intensity of the pixels comprised in each ROI is being measured in gray-scale levels, from 0 (black pixels) to 255 (white pixels), and expressed in mean ± SD through histogram analysis. Furthermore a virtual color image of the bowel can be obtained, composed of a primary color scale varying from red (maximum signal intensity) to blue (minimum signal intensity) and correlating with the numeric value of the signal intensity expressed in percentages (maximum intensity = 100%). In each patient two different regions of the color map with the highest echo-signal intensity after contrast injection, were analyzed and the mean value was used for the statistical analysis.

Within the brightest zone of the intestinal wall, which represents the highest degree of vascularization, we made two measurements per injection. The kinetic parameters (PI, TTP, RBV) calculated for both ROI were then averaged for each patient [18]. The radiologist performing the ultrasonography (grey scale and CEUS) was aware of the diagnosis of Crohn’s disease, but blinded to the patient’s present clinical, endoscopic and MRE data.

### Statistical Analysis

Categorical variables were described using frequencies and percentages. Continuous variables were summarized using medians and range.

A Receiver Operating Characteristic (ROC) analysis was performed to determine the optimal cut-off value for different CEUS variables in order to differentiate active from inactive endoscopic disease. The value of contrast enhancement at MRE and CEUS in diagnosing active luminal CD was assessed by calculating the sensitivity, specificity, positive and negative predictive values and accuracy with 95% confidence intervals (95% CI), using endoscopic disease activity as reference standard. The sensitivity and specificity of the two modalities were compared by the McNemar test. The correlations between different radiological parameters assessed by MRE and ultrasound were determined by Spearman’s nonparametric correlation coefficient. We additionally applied the plot of the difference between the values measured by the two techniques against the mean of the measurements. This method described, by Bland and Altman, allows the visualization of the concordance between two tests for different measured values, in order to examine the actual agreement more closely. The smaller the difference between the values measured by the two tests, the better the correlation [29].

P values less than 0.05 were considered to indicate statistical significance. Statistical analysis of the data was performed using the Statistical Package for the Social Sciences (SPSS 21.0).

## Results

### Study population

Over a 37-month period, 107 consecutive patients were included. The main demographic and clinical data are shown in Table 2. Two CD patients (2%) were not included in the analysis because their terminal ileum could not be visualized by B mode US and therefore no contrast assessment was done. Both patients had no active ileal disease on endoscopy.

**Table 2:** Baseline demographic and clinical characteristics of the CD patients (n = 105).

Variables	Absolute frequency (%)
Male	46(44%)
Female	59(56%)
Age [median (range)]	33 (18–65)
Disease location	
Ileal	56(53%)
Colonic	12(12%)
Ileocolonic	37(35%)
Disease behavior	
Non-stricturing/non-penetrating	65(62%)
Stricturing	34(32%)
Penetrating	6(6%)
Postoperative status (ileocecal resection)	16(15%)
Serum CRP > 5mg/l	63(60%)
Clinical remission (HBI<4)	15(14%)

The endoscopically determined location and disease behavior of all patients is described in Table 2 according to the Montreal classification. Intubation of the terminal ileum was attempted in all patients. However, 11 (10%) of the 105 patients had a stenosis (colon or ileocecal valve) disallowing endoscopic intubation of the terminal ileum. An incomplete stricture of the terminal ileum that could be passed by the endoscope was present in 19 (18%) patients. Twelve patients (11%) had a normal appearance of the terminal ileum next to an inflamed colon and these were classified as L2 according to the Montreal classification [30]. Endoscopic disease activity at the site of the terminal ileum was present in 82 (79%) of the patients.

### MR Enterography

No adverse events from MRE, performed as per protocol in all patients, were reported.

The signal intensity after administration of gadolinium was scored as normal in 27 patients (26%) and increased in 78 (74%) of the patients.

Overall sensitivity of gadolinium enhancement in detecting the presence of any active inflammation of the terminal ileum was 87%, with a specificity of 100% and an accuracy of 94% (Table 3).

**Table 3:** Statistical values for MR Enterography (MRE) and Contrast enhanced Ultrasound (CEUS) in detection of active endoscopic inflammation in the terminal ileum.

	Contrast enhancement at MRE (95% CI)	Contrast enhancement at CEUS (95% CI)
Positive true	71	82
False positive	0	1
Negative true	12	11
False negative	11	0
Sensitivity*	87% (77%-93%)	100% (96%-100%)
Specificity #	100% (73%-100%)	92% (62%-99%)
PPV	100% (95%-100%)	99% (93%-100%)
NPV	52% (31%-73%)	100% (71%-100%)
Accuracy	88% (80%-94%)	99% (94%-100%)

* P value for pairwise McNemar test was 0,01 for MRE versus CEUS

# P value for pairwise McNemar test was 1.0 for CEUS versus MRE.

**Fig 2:**
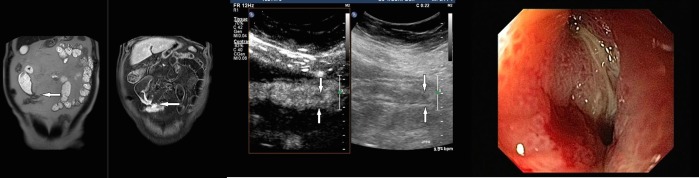
Forty- two-year-old male patient with Crohn’s disease and enteroenteral fistula of the terminal ileum. A: Coronal MR Enterography images (T2-weighted single-shot turbo spin-echo, on the left image; T1-weighted fat saturated, spectrally attenuated inversion recovery after Gadoteric acid administration, on the right image) of a patient with enteroenteral fistula and increased enhancement after contrast administration (white arrow). B: Longitudinal Contrast enhanced ultrasound of the affected ileum of the same patient (Gray scale ultrasound image on the right side; Contrast enhanced ultrasound image on the left side), showing severe wall thickening on the Gray scale ultrasound (white arrow) and strong enhancement after Sonovue administration on the Contrast enhanced ultrasound (white arrow), but no fistula. C: Endoscopic image of the same patient, with a fistula opening in the terminal ileum.

**Fig 3:**
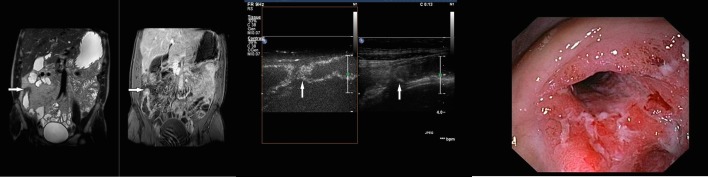
Thirty-one-year-old female patient with Crohn’s disease and stenosis of the ileocolic anastomosis after ileocecal resection. A: Coronal MR Enterography images (T2-weighted single-shot turbo spin-echo on the left image; T1-weighted fat saturated, spectrally attenuated inversion recovery after Gadoteric acid administration, on the right image) of a patient with stenosis (white arrow) and increased enhancement of the ileocolic anastomosis (white arrow). B: Longitudinal Contrast enhanced ultrasound (Gray scale ultrasound image, on the right side; Contrast enhanced ultrasound image, on the left side), of the same patient with a short stenosis of the ileocolic anastomosis (white arrow) and increased enhancement after Sonovue administration (white arrow). C: Endoscopic image of the same patient showing an ulcerative stenosis of the neoterminal ileum.

The median length of the affected terminal ileum was 7 cm (range 3–50 cm). The median measured wall thickness was 5.5 mm (range 3–13 mm). MRE detected a total of five enteroenteral fistulae (Fig 2) and sixteen cases of ileal stenosis with prestenotic dilation (Fig 3).

### Grey scale US and Contrast enhanced US

A large majority of patients (98%) were suitable for grey scale US and CEUS and no adverse effects were reported. The median value of the peak intensity (PI) was 33.5% (4–62%) and the median value of the regional blood volume (RBV) was 646.1 cm3 (18–1769,2 cm3). PI was registered after a median time to peak (TTP) of 20,5 seconds (2–42,8 seconds).

**Fig 4:**
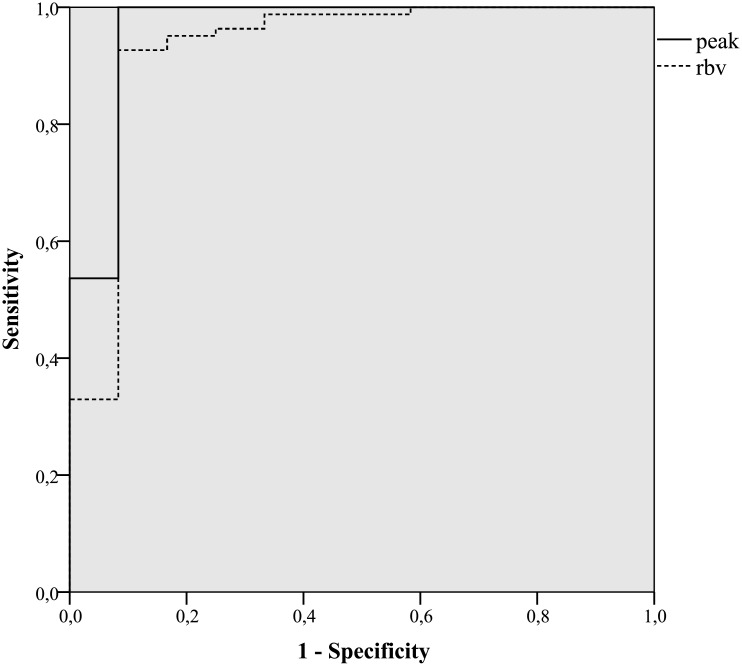
Receiver Operating Characteristic curve for performance of Contrast enhanced Ultrasound for the diagnosis of active ileitis at endoscopy. Peak intensity (solid line) and Regional Blood Volume (dotted line).

The cut-off value for PI that best depicted the presence of active endoscopic disease, indicated by the area under the ROC curve, was 10% (Fig 4). This value provided a sensitivity of 100% in detecting the presence of active inflammation at the site of the terminal ileum, with a specificity of 92% and an accuracy of 99% (Table 3).

The cut-off value for RBV that best detected active endoscopic disease was 200 cm3, achieving a sensitivity of 93%, specificity of 83% and accuracy of 88%. The area under the curve for PI was 0.96 (95%CI 0.89–1.00) and for RBV 0.93 (95% CI 0.82–1.00).

**Fig 5:**
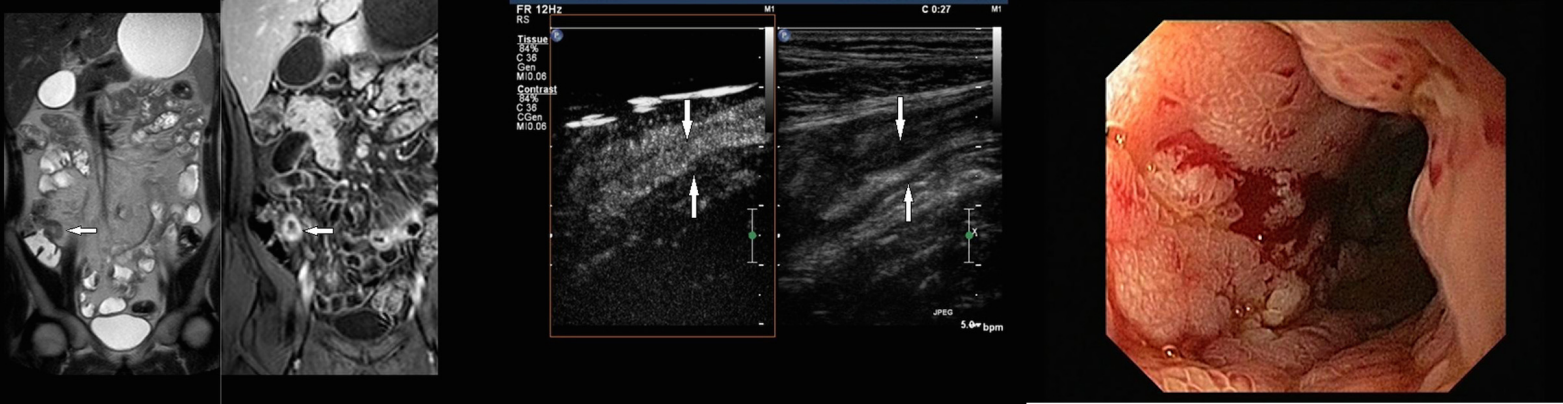
Twenty-five-year-old male patient with Crohn’s disease of the terminal ileum. A: Coronal MR Enterography images (T2-weighted single-shot turbo spin-echo on the left image; T1-weighted fat saturated, spectrally attenuated inversion recovery after Gadoteric acid administration, on the right image) of a patient with ileitis terminalis demonstrating marked wall thickening of the terminal ileum (white arrow) and mild enhancement after contrast administration (white arrow). B: Longitudinal Contrast enhanced ultrasound (Gray scale ultrasound image, on the right side; Contrast enhanced ultrasound image, on the left side), of the same patient showing wall thickening on Gray scale ultrasound (white arrow) and strong enhancement after Sonovue administration on Contrast enhanced ultrasound (white arrow). C: Endoscopic image of the same patient with an ulcerative ileitis terminalis.

The median length of the affected bowel was 6 cm (range 3–20 cm). The median measured wall thickness was 5.5 mm (range 3–13 mm) and a stratified wall appearance was detected in 53 patients (50%) (Fig 5). Furthermore, grey scale US detected stenosis of the terminal ileum with prestenotic dilation, in 11 patients.

### Correlation between MRE and US findings

**Fig 6:**
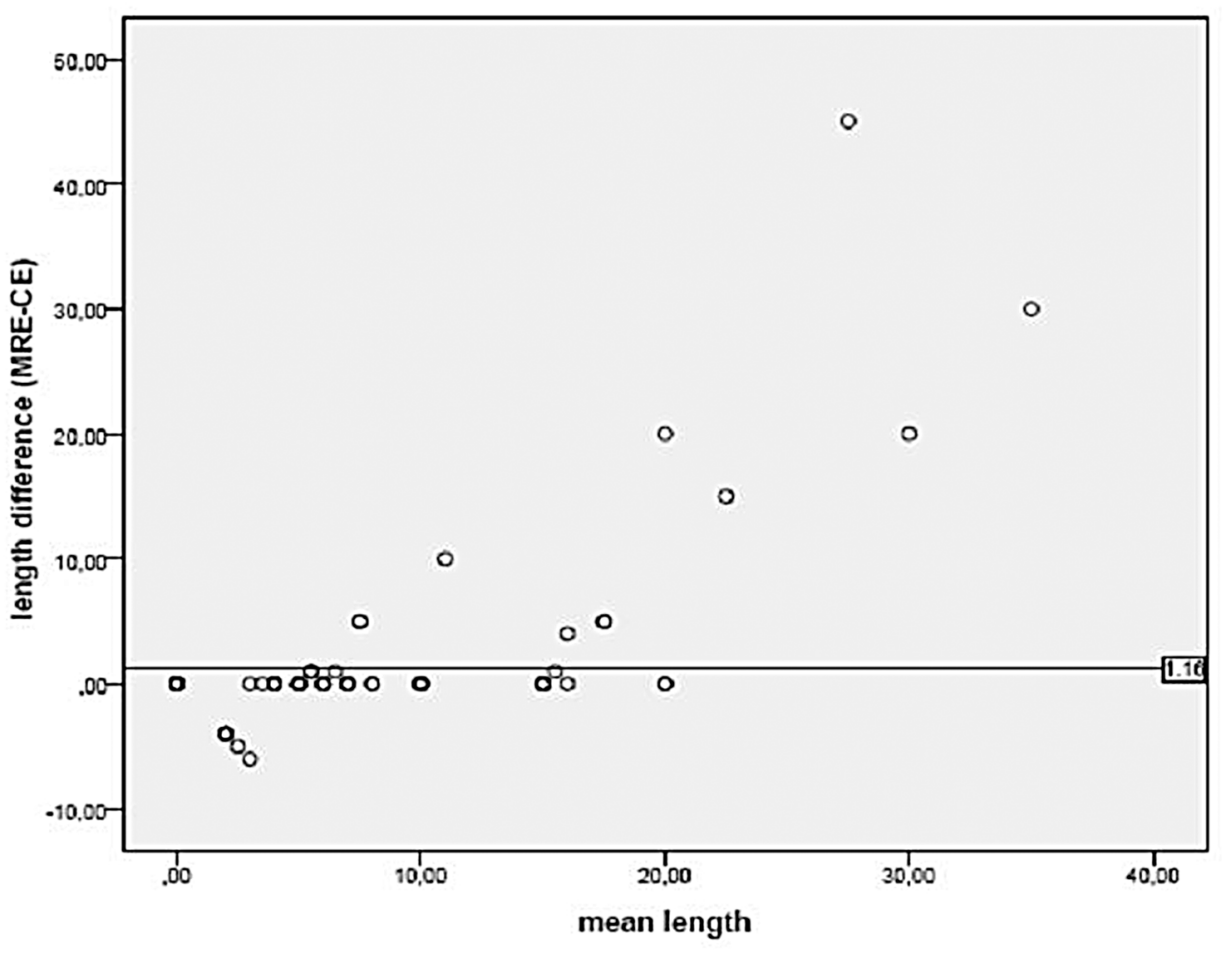
The relation between the mean length and the difference in length of the affected small bowel measured by MR Enterography and Contrast Enhanced Ultrasound.

The length of the lesion measured by MRE correlated with the measured length of the pathologic bowel wall by US (r = 0.92, p < 0.001). In order to more closely examine the agreement of measurement between the two imaging techniques, we constructed a Bland and Altman plot. There was a high agreement for diseased segments below 10 cm, but a rather poor agreement for diseased segments above 15 cm (Fig 6).

**Fig 7:**
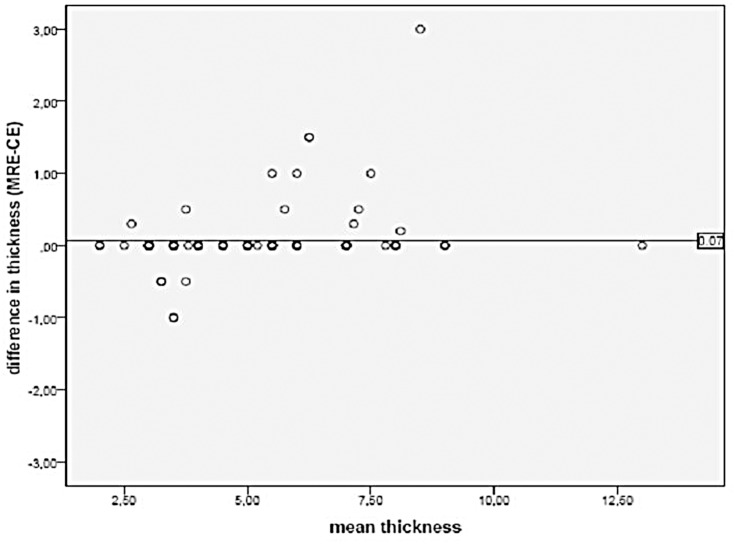
The relation between the mean wall thickness and the difference in wall thickness measured by MR Enterography and Contrast Enhanced Ultrasound.

There was also a high correlation between the wall thickness of the affected terminal ileum, between MRE and US (r = 0.97, p<0.001). When looking at the Bland and Altman plot, the agreement between the two techniques was least in patients with a wall thickness between 6 and 10 mm (Fig 7). In assessing the wall stratification and perivisceral findings, the two methods agreed in 89 and 90 out of the 105 patients, respectively.

In diagnosing stenosis, grey scale US showed a sensitivity of 63% compared to MRE (11 out of the 16 strictures similarly diagnosed). None of the five enteroenteral fistulae on MRE could be identified by US.

## Discussion

This study has demonstrated a high accuracy of CEUS combined with grey scale US for detecting mucosal disease activity in ileal CD. Grey scale US identified the (neo)terminal ileum in 105 out of 107 CD patients.

When compared to endoscopic disease activity, enhancement by means of SonoVue administration during CEUS had a high sensitivity in diagnosing active inflammation located in the terminal ileum. For assessing active intestinal inflammation, a higher accuracy for the peak intensity (PI) value than for the regional blood volume (RBV) was confirmed by CEUS-generated time-intensity curves. Regarding morphology, the US and MRE showed a high correlation for a diseased segment under 10 cm and for a wall thickness under 6 mm or above 10 mm, with slightly less accuracy for thicknesses between these two measurements. US seemed less accurate in identifying affected bowel segments longer than 10 cm. Furthermore, grey scale US identified 63% of the stenosis but none of the five enteroenteral fistulae.

In a former study, semi-quantitative findings from CEUS were found to correlate with dynamic contrast-enhanced MRE (r = 0,623, p = 0,003). CEUS and MRI findings, however, did not correlate with clinical and laboratory parameters measuring disease activity (CDAI and CRP). Therefore, the authors concluded that CEUS and MRE were not able to evaluate disease activity [23]. In a more recent study, CEUS was compared with MRE in the assessment of small bowel CD, again using clinical parameters as reference standard. In about one third of the 30 patients included, CEUS or MRE were able to detect active disease. Additionally MRE and CEUS were weakly correlated with clinically assessed disease activity (CDAI), with a Spearman’s coefficient r = 0.398 [24]. Comparison of our results with findings in the literature is difficult. Study protocols and definitions of disease activity vary considerably and standardized semi-quantitative measurements to compare outcome are lacking. In the present study, cut-off values for PI and RBV values of contrast enhancement at CEUS were calculated using ROC analysis and therefore could differ from other studies.

Increased wall microvascularization and proliferation of microvessels in the severely affected bowel of CD patients has been reported in several histopathological studies [31–33]. Furthermore, a strong relation between changes in the vascularization of the diseased bowel loop and inflammatory activity has been suggested [31,34]. Color and power Doppler ultrasonography have also been used to assess the wall hypervascularization of inflamed loops [31, 34, 35]. Findings detected by Doppler US are related to large arteries of the bowel wall and therefore this method is probably less accurate in predicting mild parietal inflammation [35]. Nonetheless, parietal contrast enhancement after intravenous gadolinium administration during MRE has been used to indicate transmural inflammation and was shown to be helpful in differentiating active from inactive CD [7, 36–38].

At present, MRE is recommended as the preferred imaging technique in CD because of its safety and its accurate, reproducible visualization of the entire small bowel tract [39]. In this study, quantitative assessment of the terminal ileum enhancement by CEUS was more sensitive in detecting active endoscopic inflammation than the qualitative assessment of enhancement after gadolinium administration during MRE. This difference may be explained by differences in the assessment technique and by the different pharmacodynamics of both contrast agents. Gadolinium chelates migrate and accumulate in the interstitium, including the fibrous tissue of chronic lesions. US contrast agents (gas microbubbles) remain in the microcirculation and degrade quickly in the vascular system. These blood-pool contrast agents theoretically lead to enhancement restricted to structures with an increased microvascularization and are not retained in the fibrous tissue [19]. This could explain a higher sensitivity of CEUS in distinguishing between active and inactive intestinal lesions.

A limitation of the diagnostic performance of US in this study was the lack of accurate detection of fistulae and abscesses that may be due to underrepresentation in this series of patients. Recently, a retrospective analysis demonstrated good accuracy of CEUS with grey scale US in diagnosing abscesses and phlegmons in CD [40]. Another limitation was that contrast enhancement at MRE was not quantitatively scored. However, quantitative scoring of contrast-uptake during MRE is not standard procedure in daily practice, being rather complex and time-consuming. Besides, several recent papers have described the value of qualitative assessment of contrast enhancement during MRE compared to endoscopic disease activity [41,42].

One of the strengths of the present series was the size of the study population, which enabled a reliable statement with respect to feasibility and sensitivity. However, the specificity may be less reliable due to the limited number of patients with a normal terminal ileum during endoscopy. Furthermore, the use of endoscopically assessed disease activity authorized an objective grading of active inflammation and therefore a reliable reference standard.

Among the cross sectional imaging techniques available for the assessment of patients with ileal CD during follow-up, the addition of CEUS to a standard grey scale US study may constitute an attractive, practical imaging modality: there is a lack of radiation exposure, it is readily available and it comes at low cost. Furthermore, three different recent studies in CD patients argue the value of CEUS in predicting outcome after treatment with immunomodulators and/or TNF blockers [43–45] and diagnosing postoperative recurrence [46]. Therefore, we believe, although not replacing MRE as an imaging technique in all patients, CEUS may well be used as an additional technique in the follow-up of patients with known ileal CD.

In conclusion, CEUS is feasible and accurate in detecting the presence and activity of CD in the terminal ileum. The performance of this imaging modality is comparable to MRE in uncomplicated ileitis, defined as a short affected terminal ileum not complicated by fistulae or abscesses. CEUS represents a valuable, easily applicable bedside tool in the follow-up of terminal ileum involvement in CD.

## Supporting Information

S1 File Supporting Information: STARD checklist. (DOC)
